# Echocardiography in Confirmed and Highly Suspected Symptomatic COVID-19 Patients and Its Impact on Treatment Change

**DOI:** 10.1155/2020/4348598

**Published:** 2020-09-16

**Authors:** Nadia Benyounes, Clélie Van Der Vynckt, Séverine Tibi, Alexandra Iglesias, Laurence Salomon, Olivier Gout, Thierry Tibi

**Affiliations:** ^1^Cardiology Unit, Rothschild Foundation Hospital, Paris, France; ^2^British Library, London, UK; ^3^Alsacienne School, Paris, France; ^4^Clinical Research Department, Rothschild Foundation Hospital, Paris, France; ^5^Neurology Department, Rothschild Foundation Hospital, Paris, France

## Abstract

**Background:**

COVID-19 interacts at multiple levels with the cardiovascular system. The prognosis of COVID-19 infection is known to be worse for patients with underlying cardiovascular diseases. Furthermore, the virus is responsible for many cardiovascular complications. Myocardial injury may affect up to 20% of the critically ill patients. However, echocardiography's impact on the management of patients affected by COVID-19 remains unknown.

**Objectives:**

To explore echocardiography's impact on the management of COVID-19 patients.

**Methods:**

This study was conducted from March 24^th^ to April 14^th^, 2020, in a single center at Adolphe de Rothschild Foundation Hospital, Paris, France. All consecutive inpatients with laboratory and/or CT COVID-19 diagnosis were included in this study. Patients' characteristics (clinical, biological, and imaging) and treatment change induced by echocardiography were collected and analyzed. Patients with and without treatment change induced by echocardiography were compared.

**Results:**

A total of 56 echocardiographies in 42 patients with highly suspected or confirmed COVID-19 were included in the final analyses. The median age was 66 (IQR 60.5–74). Echocardiography induced a treatment change in 9 cases (16%). The analyzed clinical data were not associated with any treatment change induced by echocardiography. D-dimer and Troponin levels were the only biological predictors of the induced treatment change. On echocardiography, higher systolic pulmonary arterial pressure and documented cardiac thrombi were associated with treatment changes in these patients.

**Conclusions:**

Echocardiography may be useful for the management of selected COVID-19 patients, especially those with elevated D-Dimer and Troponin levels, in up to 16% of patients.

## 1. Introduction

First seen in Wuhan, China, at the end of 2019, a novel coronavirus, named severe acute respiratory syndrome coronavirus 2 (SARS-CoV-2), became responsible for a pandemic acute respiratory disease around the world, a term coined by the World Health Organization (WHO) COVID-19, “Coronavirus Disease 2019.”

At the time of writing, COVID-19 has affected a total of 2,374,141 individuals in 185 countries and resulted in 164,716 deaths worldwide. Eighty days later, 11,850,886 patients are affected in 188 countries and 544,726 patients died [1].

It has been documented that preexisting cardiovascular disease (CVD) predisposes to COVID-19 infection and worsens COVID-19 patients' outcome [2–4].

Furthermore, at least 20% of severely ill COVID-19 patients experience cardiac injury [5]. In Chinese experience, patients with no comorbidities have a case fatality rate (CFR) of 0.9% while those with CVD, hypertension (HTN), and diabetes mellitus have CFR of, respectively, 10.5%, 6%, and 7.3% [6].

As it is sometimes difficult to differentiate cardiac from respiratory acute illness, transthoracic echocardiography (TTE) is a very useful tool in routine clinical practice. However, in the setting of COVID-19, clinicians delivering cardiovascular care are at risk of developing the illness or becoming vectors for the infection. This is especially the case for the sonographers and cardiologists who acquire TTE and mostly transesophageal (TEE) images because of a close contact, exposure, and the duration of the echocardiographic examinations. This is why, systematic TTE and TEE are not recommended in the setting of COVID-19, unless urgently or emergently necessary [7]. An initial step in reducing the number of echocardiographies in these patients would be to assess the indications rigorously. Cardiovascular considerations for patients, health care workers, and health systems during the COVID-19 pandemic have been published very recently [7].

However, in the real-life practice, we are very committed to this part of the medical practice [8–10] and we often have to adapt the recommendations and guidelines to the circumstances. This is why we had to perform TTEs/TEEs on COVID-19 patients admitted to our institution.

The objective of this study was to collect clinical, biological, and echocardiographic data of COVID-19 patients who were admitted to various departments of our institution and who underwent echocardiography, to find out the reasons of TTE/TEE examinations in these patients and to assess the impact of TTE/TEE on their management.

## 2. Materials and Methods

### 2.1. Patients and Echocardiographic Examinations

By the end of March 2020, our hospital opened dedicated departments to COVID-19 patients' care. During their hospitalization in these departments, the patients who underwent echocardiography at the discretion of their physicians were prospectively included in the study.

TTE and TEE were performed on confirmed or highly suspected COVID-19 patients admitted to our institution, by dedicated teams of one cardiologist and one nurse every time. Cardiologists and nurses were equipped with the locally advised personal protective equipment (PPE). TTEs were fast echos, all the measurements being made offline, unless emergently necessary for patients' management. Electrocardiographic monitoring was not obtained during the examination. Two-to-three-second clips were acquired of each of these views: parasternal long axis, parasternal short axis (at least at midpapillary axis), apical 4-chamber, apical 2-chamber views, and subxiphoid view (including inferior vena cava analysis throughout the respiratory cycle on M-mode imaging or clip or both).

Pulsed-wave Doppler of the mitral inflow at the level of valve leaflet tips was used to measure the peak early (E-wave) and late (A-wave) diastolic flow velocities and calculate the E/A ratio. Pulsed-wave Doppler tissue imaging was performed at the lateral and septal mitral annulus to obtain average peak longitudinal early diastolic annular (*e*′) velocity which was used to calculate the *E*/*e*′ ratio and at the tricuspid annulus. Peak velocity of the tricuspid regurgitation was determined using continuous-wave Doppler.

These clips and images allowed an analysis of the cardiac anatomy and the offline calculations (left ventricular ejection fraction, *E*/*e*' ratio, and pulmonary pressures).

A dedicated EPIQ7 ultrasound system was used, brought each time at patients' bedsides, and fully cleaned and decontaminated afterwards.

As the aim of this study was to address the impact of the echocardiographic examinations on patients' treatments, all the performed echocardiographies were included in the study.

Of note, in our hospital as in most French hospitals, echocardiographies are performed by cardiologists and not sonographers.

The very first echocardiographies prospectively performed in our hospital on confirmed or highly suspected COVID-19 patients are reported in this article.

This study was submitted to our institution's ethical comity.

### 2.2. Treatment Change

Cardiologists' recommendations and treatment modifications induced by echocardiography were prospectively collected. These interventions due to echocardiography could be the introduction of diuretics, of anticoagulation, the thrombolysis of pulmonary embolism after TEE visualization of thrombus in the pulmonary artery in an intubated instable patient, for example.

These interventions were part of the routine clinical practice.

### 2.3. Data Collection

Epidemiological, demographic, clinical, laboratory, and outcome data were prospectively extracted from electronic medical records. Treatment modifications induced by echocardiography were prospectively collected by the cardiologist himself.

### 2.4. Statistics

Continuous data were expressed as median (interquartile range [IQR]) values and were compared using the *t*-test, the Mann–Whitney *U* test, or Kruskal–Wallis H test, as appropriate. Categorical data were expressed as proportions. Pearson's chi-squared test and Fisher's exact test were used for the comparison of categorical variables, as appropriate. For all the statistical analyses, *P* < 0.05 was considered to be statistically significant. Statistical analyses were performed using STATA® software, version 13 (StataCorp LP, College Station, TX, USA).

## 3. Results

From March 24^th^ to April 14^th^, 2020, 50 TTEs, 5 TTE + TEE, and 1 TEE were performed on 42 confirmed or highly suspected COVID-19 patients admitted to our institution. In 4 cases (7.1%), nasopharyngeal swab was positive for SARS-CoV-2. In 25 cases, nasopharyngeal swab was positive for SARS-CoV-2 and chest CT scan was typical of COVID-19 (44.6%). In 22 cases (39.3%), COVID-19 was highly suspected on chest CT, which showed typical abnormalities of the disease. Finally, in 5 cases, CT showed suspect but not typical images of COVID-19 (8.9%).

Two of the included patients had three TTEs and ten patients had 2 TTEs during the study period, as indicated by the practitioner in charge. The indications for TTE/TEE are summarized in Table 1.

Table 1 describes the clinical characteristics of the included patients. The median age of the patients was 66 years and 32.1% were female. Inaugural symptoms were respiratory in 62.5% of cases and neurological in 28.6%. A total of 25% of the patients were receiving supplemental oxygen at baseline, 58.9% were intubated, and 16.1% did not need oxygen therapy at baseline. The median body mass index was 28.7 Kg/m^2^.

**Figure 1:**
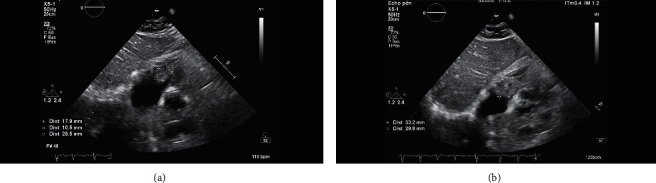
TTE, subcostal view, showing a right atrial thrombus in a COVID-19 suspected patient (deceased after discharge). The thrombus was prolapsing in the right ventricle in diastole.

**Figure 2:**
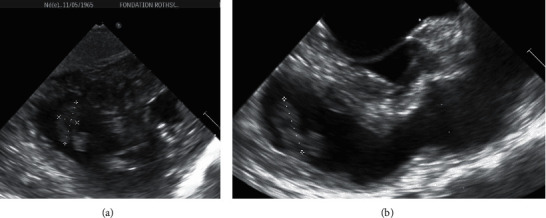
Voluminous and heterogeneous right atrial thrombus seen on TTE (a) and TEE (b). The patient had thrombolysis for severe pulmonary embolism.

Pulmonary embolism was documented in 17.9% of the whole series and cardiac thrombus in 3 patients (5.4%). Figures 1(a) and 1(b) show a right atrial thrombus seen on TTE and Figures 2(a) and 2(b) show a right atrial thrombus on TTE and TEE, respectively.

A treatment change was induced by the performed echocardiography in 9 cases (16%). These patients' characteristics and the nature of treatment change are summarized in Table 2.

Table 3 describes the biological characteristics of our patients. Among the analyzed biological parameters, D-dimer levels were significant predictors of treatment change induced by echocardiography, and so were the Troponin levels (see Table 3).

Creatinine, NT-pro BNP, fibrinogen, and blood type were not associated with echocardiography induced treatment change.

Apart from these 9 patients, there were other substantial abnormalities documented by echocardiography. In one male patient with stroke and bilateral renal infarctions, who was in atrial fibrillation, sludge was documented in the left atrial appendage by TEE. In a few other cases, TTE helped the physicians in the decision to not proceed to a vascular filling in the setting of hypotension, for example, because of high left ventricular filling pressures. However, these abnormalities did not directly change the treatment.

## 4. Discussion

In this report, the indications for TTEs were in accordance with the known cardiovascular complications of COVID-19. Hence, cardiovascular complications are becoming a major threat to this disease. After the initiation of our study, ASE Statement on Protection of Patients and Echocardiography Service Providers has been published [11].

We wish to discuss our results according to the available data in this field.

### 4.1. Prothrombotic State and Pulmonary Embolism

In 9 cases (16.1%), the indication for TTE was the suspicion or the documentation of pulmonary embolism. Indeed, pulmonary embolism has been reported in COVID-19 patients [12], and D-dimer levels have been identified as prognostic markers in infected patients in China [4, 13]. Furthermore, our results identify D-dimer levels as a marker of treatment change induced by echocardiography. Hence, the median D-dimer level was 20000 ng/ml (IQR 2690-20000) in the group with treatment change versus 2770 (IQR 1350-9330) in the group without echocardiography induced treatment change (*P*=0.0142). Of note, in 10 cases (17.9%), CT scan has documented pulmonary embolism in this study. The association between COVID-19 induced pneumonia and pulmonary embolism has been questioned [12]. Although our study did not aim to answer this question, the results are in line with a possible association between these two pulmonary pathologies.

In 2 cases, 1 confirmed and 1 suspected COVID-19 patients, a right atrial thrombus was documented by echocardiography (see Figures 1 and 2). Patient 2 had thrombolysis for threatening pulmonary embolism.

Median estimated PAPs in our patients was 38 mmHg (IQR 31-43), with a trend to higher PAPs in the patients with echocardiographic induced treatment change (*P*=0.0549).

### 4.2. Ischemic Stroke

An abnormal coagulation state has been documented in Chinese patients, 3 of whom have been described with multiple cerebral infarctions and antiphospholipid antibodies [14].

In 10 cases, the initial manifestation of COVID-19 infection in our study was ischemic stroke. In 1 case, bilateral kidney infarctions were also documented, the patient being in atrial fibrillation, under anticoagulant treatment, and having left atrial appendage sludge on TEE.

### 4.3. Left Ventricular Systolic Function and Filling Pressures

In the general population, echocardiography is a routine test that influences therapeutic decision-making [15]. Left ventricular systolic function is of major importance in COVID-19 patients' care.

The American College of Cardiology advises restricting echocardiography in COVID-19 patients with myocardial injury or elevated natriuretic peptide to those in whom echocardiography would be expected to meaningfully affect outcome [16]. In our current study, our team has accepted all the prescribed echocardiographies for COVID-19 patients during the study period, except one.

Median LVEF of our patients was 62 (IQR 60-70), and none of our patients had severe LV systolic dysfunction. Three patients have been found to have mild LV systolic dysfunction, with LVEF between 40 and 50%. One patient was known to have ischemic heart disease, a second patient had suffered from anterior and apical ST-myocardial infarction during the current hospitalization, and a third patient was admitted for multiple ischemic strokes. In this third patient, TTE at admission was suggestive of myocardial stunning. However, the poor outcome of this patient did not allow confirmation of the diagnosis. ECG, Troponin, and NT-pro BNP at admission were normal.

Echocardiographic LV filling pressures are another important issue in the management of patients with respiratory distress since cardiac participation is often questioned. In this study, when considering the cutoff of average *E*/*e*' >  14 for diastolic dysfunction in patients with normal LVEF [17], only 4 patients were identified. The first was a 74-year-old woman admitted for COVID-19 related respiratory distress. She had persistent atrial fibrillation and has been hospitalized in another hospital 4 weeks before for heart failure with preserved LVEF. The dosage of diuretics has been increased during her stay in our hospital, but we did not include this patient in the “treatment change” group. The second was a 56-year-old man admitted for multiple ischemic strokes. ECG was normal, high-sensitivity (hs) Troponin T was 17.5 pg/ml, and NT-pro-BNP was 99.0 pg/ml. He was discharged after 9 days, without complication. The third was an 82-year-old woman with documented right atrial thrombus (see Figure 2(b)). She has unfortunately deceased in rehabilitation. The last patient was a 74-year-old woman admitted for COVID-19 related respiratory distress. She had CT-documented pulmonary embolism, abnormal ECG, hs Troponin T of 11.8 pg/ml, and NT-pro BNP of 1569 pg/ml. She has been discharged on day twelve.

Indeed, LVEF as evaluated by two-dimensional echocardiography is not sufficient alone for the diagnosis and the treatment of acute heart failure. The pitfalls of this method have been largely highlighted [18]. This is why our team of cardiologists have decided to perform systematically mitral pulsed Doppler imaging and tissue Doppler imaging, before having guidelines and recommendations in the particular setting of COVID-19, so as to approach left ventricular filling pressures and help the practitioners in fluid management of their patients (vascular filling versus diuretics).

### 4.4. Cardiac Injury

Cardiac injury is frequent in COVID-19 patients and associated with a higher risk of in-hospital mortality [5]. Recently, COVID-19 related myocarditis, confirmed by multimodality imaging, was described in a 21-year-old female patient [19]. Triggered COVID-19 Takotsubo syndrome has also been reported in an 83-year-old woman [20]. Acute myocarditis presenting as a reverse Takotsubo syndrome was also reported in a COVID-19, 43-year-old woman, with both cardiac magnetic resonance imaging and endomyocardial biopsy characterization [21].

There are various mechanisms for hs Troponin elevation in COVID-19 patients [22]. For now, the American College of Cardiology does not recommend Troponin measurement, unless the diagnosis of acute myocardial infarction is being considered [16]. However, it could be useful in determining high-risk patients, for more intensive surveillance and support [23]. In our study, only 3 patients did not have at least one Troponin measurement. The median hs Troponin T was 101.5 (IQR 30.3–111.2) in patients with a treatment change in relation to echocardiography and 20.3 (IQR 11.6–43.5) in the others. The difference did nearly reach statistical significance (*P*=0.0506).

### 4.5. Body Mass Index

The median BMI of the whole series was 28.7 (25.30–30.15) (see Table 1), and there was a trend to a higher BMI in patients with more severe illness, with mean BMI (SD) of 26.65 Kg/m^2^ (4.52) in the patients who did not require oxygen therapy (*N* = 9), mean BMI of 27.13 (3.60) in the patients requiring oxygen therapy (*N* = 14), and mean BMI of 28.88 Kg/m^2^ (4.35) in the intubated patients. However, these last analyses were exploratory, since our study was not designed for that, and the difference did not reach statistical significance (*P*=0.2374).

### 4.6. Observers' Safety

In our experience, PPE has proved to be effective. Among the two cardiologists and the three nurses who participated in the study, none had COVID-19 symptoms. Furthermore, all the five were tested in June 2020. Their serological investigations were negative.

While writing this manuscript, a helpful framework for addressing cardiovascular complications associated with COVID-19 has been published [23]. It recommends Troponin evaluation in all confirmed COVID-19 patients who require hospitalization. Our results are in line with it since only 7% of our patients did not have Troponin evaluation. Furthermore, our results confirm the usefulness of TTE in the management of COVID-19 patients with elevated cardiac biomarkers and D-dimer. Point-of-care ultrasound (POCUS) was not yet developed in our institution. However, echocardiographies were fast echos in all the patients, unless an abnormal finding was documented. In this case, the echocardiography was complete.

### 4.7. Study Limitations

Our study has a modest sample size to assess the impact of echocardiography on COVID-19 patients' management and there was no power calculation. However, we are still including patients.

Initial data on heart consequences of this dramatic disease have been rapidly published since February 2020. However, we believe that this study is, as of this date, the first study describing a population of COVID-19 patients who underwent echocardiography and evaluating the impact of this echocardiography on the management of these patients, in correlation with clinical, biological, and echocardiographic data.

Finally, how was the treatment change directly prompted by echocardiography could be questioned. However, this study was exclusively driven by practical routine clinical practice. Hence, echocardiography was performed at the discretion of the practitioner in charge of the patient, in order to respond to one or more of his unresolved questions regarding patient's cardiac condition. Subsequent decisions regarding the treatment were taken jointly by the practitioner and the cardiologist, depending on the results of the echocardiography.

## 5. Conclusion

The majority of our patients had preserved LVEF and normal LV filling pressures while pulmonary embolism was documented in 17.9% of cases.

We found 16% of treatment change induced by echocardiography, and D-dimer and Troponin levels were predictive of this treatment change.

We believe that these findings may be useful in general practice for selecting the patients who would more benefit from echocardiography, especially in the hospitals and the countries where cardiologic and echocardiographic resources are rare, in this uncertain period, and the fear of a second wave.

Finally, none of the practitioners involved in the study had symptoms of COVID-19 and all their serological investigations were negative.

Further research on a larger scale would be needed to confirm our findings.

## Figures and Tables

**Table 1:** Clinical and imaging characteristics of the patients included.

Characteristics	All TTE/TEE patients (*n* = 56)	According to treatment change		*P* value
		TTE/TEE patients with treatment change induced by the TTE *N* = 9 (16%)	TTE/TEE patients without treatment change induced by the TTE *N* = 47 (84%)	
Age, median (IQR), *y*	66 (60.5–74)	64 (59–74)	67 (62–74)	0.78
Female, *N*(%)	18 (32.1)	3 (33.3)	15 (31.9)	0.93
COVID-19 diagnosis, *N*(%) [1] positive nasopharyngeal swab [2] typical chest CT scan [3] atypical pneumonia on CT scan	[1]: 4 (7.1) [1 + 2]: 25 (44.6) [2]: 22 (39.3) [3]: 5 (8.9)	[1]: 0 [1 + 2]: 5 (55.6) [2]: 3 (33.3) [3]: 1 (11.1)	[1]: 4 (8.5) [1 + 2]: 20 (42.6) [2]: 19 (40.4) [3]: 4 (8.5)	0.75
Inaugural symptoms^$^, *N*(%)	Neuro.: 16 (28.6) Resp.: 35 (62.5) Viral: 3 (5.3) COVID typical: 2 (3.6)	Neuro.: 2 (22.2) Resp.: 6 (66.7) Viral: 1 (11.1) COVID typical: 0	Neuro.: 14 (29.8) Resp.: 29 (61.7) Viral: 2 (4.3) COVID typical: 2 (4.3)	0.75
Symptoms' severity, *N*(%) [1] no need for oxygen [2] need for oxygen therapy [3] intubated	[1]: 9 (16.1) [2]: 14 (25) [3]: 33 (58.9)	[1]: 0 [2]: 3 (33.3) 3 : 6 (66.7)	[1]: 9 (19.1) [2]: 11 (23.4) [2]: 27 (57.5)	0.35
Body area, median (IQR), m^2^	1.895 (1.83–1.97)	1.92 (1.83–2.06)	1.89 (1.83–1.97)	0.35
BMI, median (IQR), Kg/m^2^	28.7 (25.30–30.15)	29.6 (29.1–31.3)	28.1 (24.8–29.8)	0.08
Diabetes, *N*(%)	24 (43.6)	3 (33.3)	21 (45.7)	0.50
Hypertension, *N*(%)	35 (63.6)	7 (77.8)	18 (60.9)	0.34
Smoking, *N*(%)	4 (7.7%)	0	4 (8.9)	0.41
Alcohol, *N*(%)	4 (7.8)	0	4 (9.1)	0.41
Known cardiovascular disease, *N*(%)	16 (28.6)	2 (22.2)	14 (29.8)	0.65
Evolution^#^, *N*(%) 1 discharge (home or rehabilitation) 2 deceased 3 still in hospital at the time of writing	1 : 20 (35.7) 2 : 5 (8.9) 3 : 31 (55.4)	1 : 1 (11.1) 2 : 1 (11.1) 3 : 7 (77.8)	1 : 19 (40.4) 2 : 4 (8.5) 3 : 24 (51.1)	0.24
Patients with TTE/TEE, *N*(%)	TTE: 50 (87.5) TTE + TEE: 5 (10.7) TEE: 1 (1.8)	TTE: 7 (77.8) TTE + TEE: 2 (22.2) TEE: 0	TTE: 42 (89.4) TTE + TEE: 4 (8.5) TEE: 1 (2.1)	0.44
Indications for TTE/TEE, *N*(%) 1 pulmonary embolism: suspicion or evaluation/pulmonary pressures 2 left ventricular filling pressures 3 troponin *T* hs elevation 4 exclude infectious endocarditis 5 left ventricular systolic function/hemodynamic 6 ischemic stroke: seek cardiac source of embolism 7 other (valvular prosthesis, renal embolism, etc.)	1 : 9 (16.1) 1 + 2+5 : 6 (10.7) 1 + 4+6 : 1 (1.8) 1 + 5+6 : 1 (1.8) 2 : 6 (10.7) 1 + 2+5 + 7 : 1 (1.8) 3 : 2 (3.6) 4 : 4 (7.14) 5 : 11 (19.6) 1 + 5 : 2 (3.6) 6 : 8 (14.3) 6 + 7 : 1 (1.8) 7 : 4 (7.14)	1 : 0 1 + 2+5: (11.1) 1 + 4+6 : 0 1 + 5+6 : 1 (11.1) 2 : 3 (33.3) 1 + 2+5 + 7 : 0 3 : 1 (11.1) 4 : 0 5 : 2 (22.2) 1 + 5 : 0 6 : 1 (11.1) 6 + 7 : 0 7 : 0	1 : 9 (19.15) 1 + 2+5 : 5 (10.6) 1 + 4+6 : 1 (2.1) 1 + 5+6 : 0 2 : 3 (6.4) 1 + 2+5 + 7 : 1 (2.1) 3 : 1 (2.1) 4 : 4 (8.5) 5 : 9 (19.15) 1 + 5 : 2 (4.3) 6 : 7 (14.9) 6 + 7 : 1 (2.1) 7 : 4 (8.5)	0.18
CT/TEE documented PE, *N*(%)	Yes: 10 (17.9) No: 16 (28.6) Not assessed: 30 (53.5)	Yes: 2 (22.2) No: 3 (33.3) Not assessed: 4 (44.4)	Yes: 8 (17.0) No: 13 (27.7) Not assessed: 26 (55.3)	0.83
LVEF^*∗∗*^ on TTE	>50%: 51 (91.1) 40–50%: 5 (8.9)	>50%: 8 (88.9) 40–50%: 1 (11.1)	>50%: 43 (91.5) 40–50%: 4 (8.5)	NS
LVEF on TTE, median (IQR), (%)	62 (60–70)	64 (52–76)	62 (60–70)	0.90
E/A (transmitral flow), median (IQR)	0.8 (0.7–1)	0.75 (0.6–1.0)	0.8 (0.8–0.9)	0.50
E/Ea on TTE, median (IQR)	8.8 (6.5–11.8)	8.5 (6.7–10.4)	8.9 (6.5–11.8)	0.83
Systolic PAP, median (IQR), mmHg	38 (31–43)	40 (38–55)	36 (30–43)	**0.0549**
Pericardial effusion, *N*(%)	2 (3.6)	0	2 (4.3)	0.53
Cardiac thrombus (TTE or TEE), *N*(%)	3 (5.4)	3 (33.3)	0	**≤0.001**

IQR: interquartile range; *y*: years, CT: computed tomography; PE: pulmonary embolism; PAPs: echocardiographic systolic pulmonary arterial pressure. ^$^Inaugural symptoms: Neuro.: neurological; Resp.: respiratory; Viral: nonspecific viral syndrome; COVID: typical signs and symptoms of COVID-19. ^#^Evolution: 1 discharge (to home or rehabilitation); 2 deceased; 3 still in hospital on 17/04/2020 (at the date of writing this manuscript). ^*∗∗*^None of the patients had LVEF <40% in this study.

**Table 2:** Clinical, biological, and echocardiographic parameters of the 9 patients with treatment change induced by echocardiography.

Patients	1	2	3	4	5	6	7	8	9
Age (years)	56	54	82	76	64	74	62	59	66
Gender	M	M	F	M	F	M	M	M	F
COVID-19 diagnosis	Chest CT	Chest CT + NP swab	Pneumonia	Chest CT + NP swab	Chest CT + NP swab	NP swab	Chest CT + NP swab	Chest CT	Chest CT
Severity of symptoms	Intubated	Intubated	Need for oxygen	Intubated	Intubated	Intubated	Need for oxygen	Need for oxygen	Intubated
Initial symptoms	Ischemic stroke	Resp. distress	Stroke + Resp. distress	Resp. distress	Resp. distress	Resp. distress	Resp. distress	Resp. distress	Viral syndrome
Indication for TTE/TEE	Stroke + hemodynamic	LV function/hemodynamic	Stroke + hemodynamic	LV FP	LV FP	Hemodynamic	ACS ST-	LV function/hemodynamic	Resp. distress
LVEF (%)	>50	>50	52	76	83	>50	47	59	69
E/Ea	7	NA	16	9.2	8.5	NA	10.4	5.1	6.7
RV dilatation	0	1	0	0	0	1	0	0	0
PAPs (mmHg)	35	80	NA	40	39	55	NA	42	38
Echo finding inducing the modification	Inferior wall MI	RA thrombus	RA thrombus	Not elevated LV FP	Low LV FP	Right pulmonary artery thrombus	Akinesia	Aortic aneurysm	Normal LV, low LV FP
Treatment change	DAPT	Thrombolysis (PE)	AC	Stop diuretics	Fluid administration	NFH	DAPT	ß bloq	Stop IV nitrates
Known CVD	No	No	Yes	Yes	No	No	No	No	No
HTN	No	Yes	Yes	Yes	Yes	Yes	No	Yes	Yes
Diabetes	Yes	No	No	No	No	Yes	Yes	No	No
Body area (m^2^)	2.06	2.35	1.87	2.19	1.75	1.97	1.92	1.83	1.69
BMI (Kg/.m^2^)	29.3	34.0	26.7	31.3	34.9	29.6	27.0	31.2	29.1
Hs Troponin T (pg/ml)	776,9	9.3	105.2	101.5	53.6	112.2	172.8	30.3	8.9
NT-pro BNP (pg/ml)	750	206	15652	219	2808	6119	4038	339	370
D-dimer (ng/ml)	20000	5670	20000	2690	1550	20000	2590	20000	20000
Fibrinogen (g/l)	1.2	8	5.7	4.4	12	5.2	12	7.2	8
Blood type	O+	AB+	NA	B−	NA	O+	O+	O+	A−
Evolution^#^	Discharge	Still in hospital (ECMO)	Deceased	Still in hospital	Still in hospital	Still in hospital	Still in hospital	Still in hospital	Still in hospital
Pulmonary embolism	Not assessed	Yes	No	Not assessed	No	Yes	Not assessed	No	Not assessed

F: female; M: male; LVEF: left ventricular ejection fraction; NA: not available; RV: right ventricular; HTN: hypertension; chest CT: high CT probability of COVID-19 infection; DAPT: dual antiplatelet therapy; NP swab: positive nasopharyngeal swab; MI: myocardial infarction; PE: pulmonary embolism; AC: anticoagulant (add or modify anticoagulation regimen); RA: right atrial; Resp. distress: respiratory distress; NFH: nonfractioned heparin; CVD: cardiovascular disease; FP: filling pressures; ß bloq: beta bloquers. ^#^Evolution: 1 discharge (to home or rehabilitation); 2 deceased; 3 still in hospital on 17/04/2020 (at the date of writing this manuscript).

**Table 3:** Biological characteristics of the patients included.

Characteristics	All TTE/TEE patients (*n* = 56)	According to treatment change		*P* value
		TTE/TEE patients with treatment change induced by the TTE *N* = 9 (16%)	TTE/TEE patients without treatment change induced by the TTE *N* = 47 (84%)	
Creatinine, median (IQR), *µ*mol/l	80 (58.5–124)	90 (77–126)	74 (58–122)	0.4961
Hs Troponin T, median (IQR), pg/ml	21 (11.8–57)	101.5 (30.3–111.2)	20.3 (11.6–43.5)	**0.0506**
NT-pro BNP, median (IQR), pg/ml	628 (177–2530)	750 (339–4038)	552.5 (155.5–2138.5)	0.3197
D-dimer, median (IQR), ng/ml	2970 (1130–11620)	20000 (2690–20000)	2770 (1350–9330)	**0.0142**
Fibrinogen, median (IQR), g/l	7.84 (5.72–10.2)	7.15 (5.17–7.95)	8.64 (5.89–10.2)	0.4111
Blood type, N (%)	O+: 17 (46.0) A+: 9 (24.3) A−: 2 (5.4) AB+: 2 (5.4) B+: 7 (18.9)	O+: 4 (57.1) A+: 0 A−: 1 (14.3) AB+: 1 (14.3) B+: 1 (14.3)	O+: 13 (43.4) A+: 9 (30.0) A−: 1 (3.3) AB+: 1 (3.3) B+: 6 (20.0)	0.2900

NT-pro BNP : N-terminal pro B natriuretic peptide; Hs Troponin T: high-sensitivity cardiac Troponin T.

## Data Availability

The underlying data may be obtained from the corresponding author.

## Abbreviations

COVID-19: Coronavirus disease 2019
CRF: Case fatality rate
CVD: Cardiovascular disease
*E*/*e*': Mitral valve *E* velocity divided by mitral annular *e*' velocity
HTN: Hypertension
Hs Troponin: High-sensitivity Troponin
LVEF: Left ventricular ejection fraction
PAPs: Pulmonary arterial systolic pressure
PPE: Personal protective equipment
TEE: Transesophageal echocardiography
TTE: Transthoracic echocardiography.
